# Virtual Reality for Surgical Planning – Evaluation Based on Two Liver Tumor Resections

**DOI:** 10.3389/fsurg.2022.821060

**Published:** 2022-02-28

**Authors:** Anke V. Reinschluessel, Thomas Muender, Daniela Salzmann, Tanja Döring, Rainer Malaka, Dirk Weyhe

**Affiliations:** ^1^Digital Media Lab, University of Bremen, Bremen, Germany; ^2^University Hospital for Visceral Surgery, Pius-Hospital Oldenburg, Carl Von Ossietzky University Oldenburg, Oldenburg, Germany

**Keywords:** virtual reality, 3D models (three dimensional), surgery planning, human-computer interaction, user study

## Abstract

**Purpose:**

For complex cases, preoperative surgical planning is a standard procedure to ensure patient safety and keep the surgery time to a minimum. Based on the available information, such as MRI or CT images, and prior anatomical knowledge the surgeons create their own mental 3D model of the organ of interest. This is challenging, requires years of training and an inherent uncertainty remains even for experienced surgeons.

**Goal:**

Virtual reality (VR) is by nature excellent in showing spatial relationships through its stereoscopic displays. Therefore, it is well suited to be used to support the understanding of individual anatomy of patient-specific 3D organ models generated from MRI or CT data. Utilizing this potential, we developed a VR surgical planning tool that provides a 3D view of the medical data for better spatial understanding and natural interaction with the data in 3D space. Following a user-centered design process, in this first user study, we focus on usability, usefulness, and target audience feedback. Thereby, we also investigate the individual impact the tool and the 3D presentation of the organ have on the understanding of the 3D structures for the surgical team.

**Methods:**

We employed the VR prototype for surgical planning using a standard VR setup to two real cases of patients with liver tumors who were scheduled for surgery at a University Hospital for Visceral Surgery. Surgeons (N = 4) used the VR prototype before the surgery to plan the procedure in addition to their regular planning process. We used semi-structured interviews before and after the surgery to explore the benefits and pitfalls of VR surgical planning.

**Results:**

The participants used on average 14.3 min (SD = 3.59) to plan the cases in VR. The reported usability was good. Results from the interviews and observations suggest that planning in VR can be very beneficial for surgeons. They reported an improved spatial understanding of the individual anatomical structures and better identification of anatomical variants. Additionally, as the surgeons mentioned an improved recall of the information and better identification of surgical relevant structures, the VR tool has the potential to improve the surgery and patient safety.

## 1. Introduction

When surgeons prepare for complex liver surgery, e.g., removing tumors (tumor resection) from the liver, they plan their procedure beforehand. Even though all surgeries follow a standard operation procedure, a patient individual plan is necessary for every tumor resection. The anatomical structures of every person deviate and tumors are always in different places and to a different extent. The spatial relationships of blood vessels and tumor tissue are very important. The distance between these two can be crucial for the decision on how to approach the operation and, more importantly, if the tumor can be removed at all. Therefore, planning the intervention is a critical stage in the decision making on how to treat the patient (1).

The surgical planning is based on MRI or CT images which represent the 3D anatomical structures as a stack of 2D gray scale images. Interpreting these images to determine where tumors are located and how they are positioned in relation to other anatomical structures requires years of experience and an inherent uncertainty still remains. In addition, reconstructing the 3D representation that is required to understand the anatomical structures and their relations from the stack of 2D images is done by the surgeons in their minds. This process requires extensive training and is often challenging even for experienced surgeons. Furthermore, as each surgeon builds a 3D representation in their own mind, agreements between surgeons are mostly discussed without a shared visual image of the 3D structures.

Existing software for surgical planning is almost always based on the 2D MRI/CT images displayed on a standard monitor. Planning the spatial process of the operation based on 2D images leads to the software only supporting limited functions such as measuring distances on a 2D plane or drawing circles on individual images to indicate important or critical structures. In addition, these tools often have unintuitive or complex controls and surgeons lack the time to put additional effort into learning these tools.

In recent years a lot of research has been done to create 3D models based on the image data (2–5). Through segmentation of the images, the individual anatomical structures are defined and combined into a polygonal model. Such models could help to overcome the challenges of the planning process but are by far not used in the standard procedure.

Virtual reality (VR) could be especially helpful to view and interact with these 3D models as it provides the user a stereoscopic 3D view of the model and enables the correct perception of depth. This could lead to a better spatial understanding of the anatomical structures and their relations (6). While augmented reality (AR) provides similar benefits, VR is especially helpful because it allows the users to immerse themselves into a dedicated planning environment without distractions from the surroundings. In contrast to this, AR is especially useful in intraoperative settings, as the user, i.e., surgeon, can still experience the real world directly. This has been demonstrated for example by Gasques et al. (7). In addition, VR can provide a sense of embodiment, referring to the user's sense of self-location and sense of agency (8) through intuitive controls to view the model just by head movement as well as the possibility to interact with objects through virtual hands. This form of interaction refers to the concept of *natural user interfaces* which are described as interfaces that provide an intuitive interaction style and express naturalness which refers to the way users interact with and feel about the product (9). In the context of surgical planning, this form of interaction can empower users to better orient and express themselves in 3D space and understand spatial relationships. This can be especially helpful when it comes to the tasks that are performed in surgical planning, e.g., marking 3D structures or indicating cuts, which are inherently spatial tasks.

Several research prototypes were presented to support viewing imaging data and planning surgeries. Concept ideas for viewing 3D renderings stereoscopically were presented as early as 1995 by Peters (10) and 1996 by Robb et al. (11). With improving hardware more prototypes viewing 3D models on 2D screens were presented in research, differing in function and interaction technique. Lee et al. (12) and De Paolis et al. (13), for example, combined 3D models with gesture input on a 2D screen. In neurosurgery, one example by Lo et al. (14) uses a 3D projector with polarization to view a model in 3D and as an interaction method a 3D mouse is used. Haptic input devices are also used as input devices like in the study by Olofsson et al. (15) and Eagleson et al. (16). Bornik et al. (17) presented a special input device to manipulate medical data on a touch screen and in VR. Sousa et al. (18) developed a VR reading room for MRI and CT images using touch as an interaction modality. Although the idea of viewing medical images or even 3D models created based on the medical images in a stereoscopic fashion is researched for decades already, commercial products are slowly incorporating viewing 3D images on 2D screens. The features available by the CANON software imply that their software can create some kind of 3D visualization^1^. With the announcement of the Microsoft HoloLens 2^2^, Siemens announced “syngo.via Cinematic Rendering^3^” which enables the user to view a volumetric rendering of MRI and CT images in augmented reality. They list the following possible interactions: “Enlarge, Zoom, and Rotate.” This shows, that a powerful VR planning tool, like the one presented in the evaluation which incorporates the advances in research, has not been used in a real application scenario to the best of our knowledge. Therefore, using a user-centered approach during design and evaluation, we aim to create valuable insights in the application of such tools in a realistic setting.

In this first evaluation, we employ a VR prototype for surgical planning to two real cases of patients with liver tumors who were scheduled for surgery. Four surgeons used the prototype as a sophisticated pilot study before the surgery to plan the procedure in addition to their regular planning process. We used individual and group interviews with the surgeons before and after the surgery to explore the benefits and pitfalls of VR surgical planning. We found that all surgeons valued that the tool provides easy and immediate access to the patient's individual anatomy and, therefore, enables them to understand the spatial anatomy and identify surgical relevant anatomical variants faster. They described the tool as easy to use and directly wanted to integrate it into their standard workflow. By conducting evaluations of the prototype under real conditions, we gain insights on how VR as a technology and related concepts of embodiment, natural and spatial interaction benefit surgeons in their daily work, how it possibly improves the safety of surgeries, and how this technology could be integrated into medical applications.

## 2. Case Description

**Figure 1:**
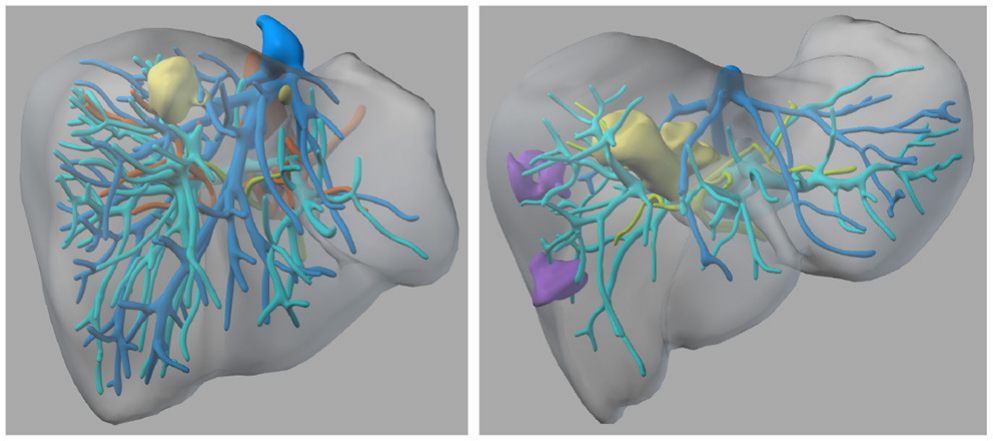
The 3D models of the two liver cases—left: Case 1; right: Case 2—Color coding: yellow = tumor tissue, blue and cyan = veins, green = biliary tracts, orange = arteries, purple = suspected lesions.

The presented evaluation comprises two anonymized cases of liver tumors, which were scheduled for surgery at the University Hospital for visceral surgery, PIUS-Hospital Oldenburg, Germany. The study received ethical consent from the medical ethics board of the University of Oldenburg and the patients agreed to participate in a study using extended imaging to plan their surgeries using our VR tool. Both were under the premise, that the surgeries are performed using standardized approaches and methods. Case 1 (C1) had two tumors, which were located relatively close to the surface of the liver (refer to Figure 1). Case 2 (C2) had one large tumor that was located in the right center of the liver close to the veins and arteries, which are essential for the blood supply of the liver, and two suspected tumor masses in the right liver part (refer to Figure 1 right side). Both patients received an MRI scan with a contrast agent of the liver for diagnostics and to provide the surgeons with preoperative information about the spatial relationships and structures. The hospital did an MRI with a contrast agent instead of CT, because it has a higher sensitivity and specificity and it is endorsed by the clinical practice guidelines in Germany. We invited the surgical team before they performed the surgery to additionally plan the surgery with our VR tool. With our approach, we followed the standard operating procedure, in which the surgeons prepare the surgery first on their own, and then the operating team has a short team time out to have a common ground (normally just before they start the surgery).

## 3. Virtual Reality Surgery Planning Tool

**Figure 2:**
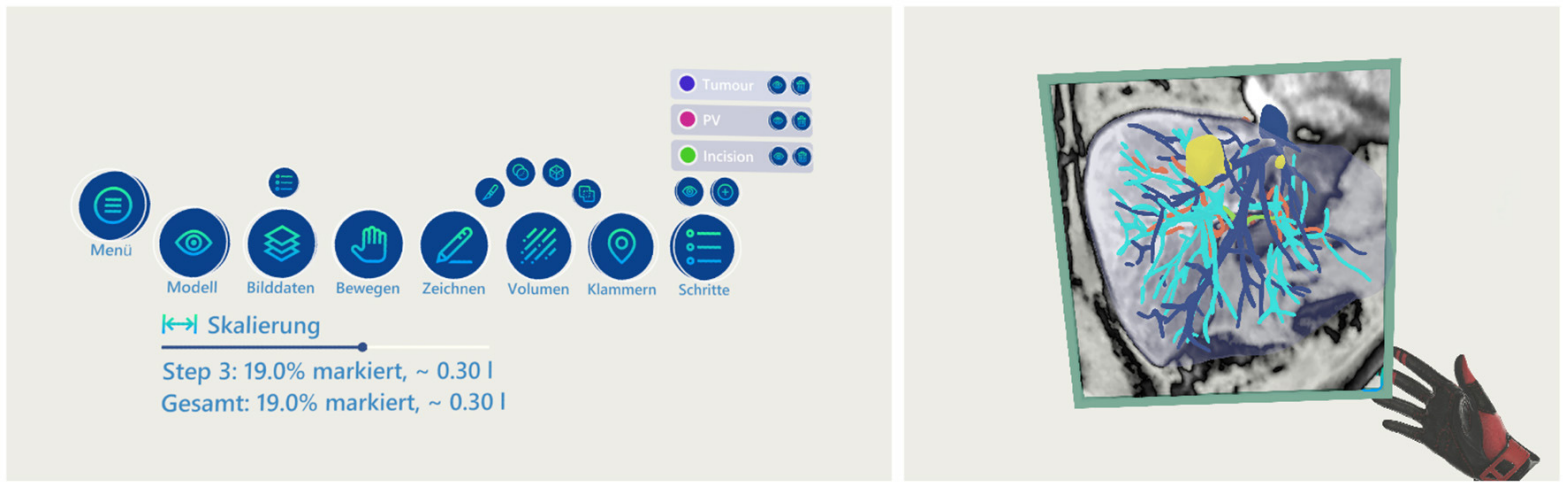
The menu and the frame functionality—left: the menu showing all available functions; right: the frame showing an overlay of the MRI images at the given intersection of the model.

In order to give surgeons the possibility to plan an operation in VR, we developed a prototype, which is focused on a patient-specific 3D model of the diseased organ. It provides different tools and interactions for the planning informed by a user-centered approach. The virtual models were created based on anonymised MRI images of the patients. The images were annotated by (medical) radiological technologists using dedicated planning software and combined into a polygonal model. For further details of the process refer to Schenk et al. (2), Marro et al. (3), and Reinschluessel et al. (19). The anatomical structures of the model, e.g., tumors, arteries, veins are colored according to anatomy textbooks (refer to Figure 1). The model is positioned in the center of an empty virtual room in mid-air at a comfortable height. In addition to the model, an image frame is present in the room that shows the MRI images at the corresponding position of the frame (refer to Figure 2 right side). It can be used to view one set of MRI images, which the model is based on. The user can interact with the model and the image frame through direct manipulation by grabbing and then moving and turning the objects with the VR controller.

A menu consisting of 3D buttons is present in mid-air to control some of the functions of the application (refer to Figure 2 left side). The interaction with the menu can be done from a distance through a pointer by using the trigger button of the VR controller. The menu has buttons for general controls, e.g., to hide/show the organ model or the image plane, as well as to switch to different tools. Furthermore, the menu provides a control element for scaling the model between the original size in the human body (100%) up to 300%.

**Figure 3:**
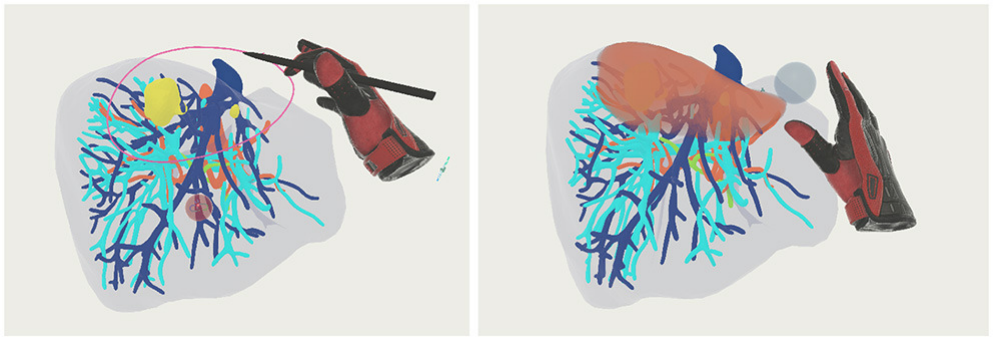
Visualization of the line drawing, clips and volume marking functionality—left: one clip and a line are added to the model; right: using the ball shaped tool at the hand the red volume was marked.

Three tools are provided to support the planning of the operation and record the actions that should be made. First, the drawing tool can be used to draw lines in the 3D space, indicating where cuts should be made or marking areas of interest (refer to Figure 3). When the drawing tool is selected a pen is displayed in the hand of the user. By pressing the trigger of the controller a line is drawn in 3D space at the tip of the pen until the trigger is released. Second, the volume tool can be used to mark the volume of the model, indicating the parts of the organ that should be removed in the surgery. The user can select from four shapes to be used for marking: a scalpel (only the front part is used for marking), a sphere, a box, and a plane. When selected, the shape is displayed in front of the hand of the user and when pressing the trigger of the controller, the overlapping parts of the shape with the organ are marked. The marked volume is represented as a colored cloud inside the model (see Figure 3). The percentage that the marked volume takes up of the whole model is calculated and presented to the user below the menu. When the user selects the drawing or volume tool, the default interaction of grabbing and moving objects is replaced with the specific drawing or marking functionality. The menu has a button to switch back into the default mode where the objects of the scene can be moved. Last, the clip tool provides the ability to add and position clips in the model. Clips are used as indicators where blood vessels should be clipped during the operation. They are represented as small red spheres with a clip icon inside and can be moved similarly to the model and image frame by grabbing and moving them with the controller. All actions of the tools are associated with steps. The menu gives an overview of the current steps and new steps can be created. Each step can be named, hidden (associated drawings/volume/clips are set invisible), and deleted. Each step has a specific color. Actions from the drawing and volume tools are colored in the respective color of the step they are associated with.

The VR setup consisted of an HTC Vive Pro and a computer with an Intel i7 processor and an Nvidia 2080 TI graphics card. Room-scaled tracking was used with an approximate area of 3 × 3 m. The application was controlled with one Vive controller, which was held in the dominant hand of the participant. All interactions could be performed with the index finger by pressing the trigger button on the controller. The application was designed around this one-button-style interaction and the concept of flat menus in order to keep it as simple as possible and make it easy to learn in a short time.

## 4. Methods

Four participants (1 women, 3 men) were recruited at the University Hospital for visceral surgery, PIUS-Hospital Oldenburg. All participants have experience in general and/or visceral surgery. The participants are between 34 and 54 years old (mean = 45.25 years, median = 46.5 years, SD = 8.54). P1 is a superintendent with 25 years of experience in the field and has performed more than 100 liver surgeons as lead surgeon. P2 is a fellow with 11 years of experience in the field and assisted more than 100 liver surgeries. P3 is a resident with 10 years of surgical experience and assisted in ten liver surgeries so far. P4 is a consultant with 17 years of experience and performed in more than 100 liver surgeries. All of the participants have previous experience with 3D models and VR, not necessarily in combination. Yet, the standard procedure at the University Hospital for Visceral Surgery, PIUS-Hospital Oldenburg relies on 2D data and previous the experience with surgical planning using 3D models is sparse. The two discussed cases have an overlap with respect to the team members as three participants represent a surgical team. Due to the pandemic situation in 2020, one team member (P1) of C1 had to be replaced on short notice after phase two out of three. P4 replaced the member, and we did an interview and planning just before the actual surgery. The participants did not receive any form of compensation for their participation.

The study had three phases and each phase was accompanied by semi-structured interviews. The first phase started with an interview, then the participants got an introduction to the application using an exemplary case. The introduction was skipped for the second case, as all participants were familiar already with the tool from C1. Nevertheless, we asked them if they needed a refresh of the tool description. When the participants felt confident with the application, they got the 3D model of the patient to be operated on. They were instructed to plan the surgery using the available tools and to follow the think-aloud protocol (20). After the participants finished the planning, the second part of the interview with questions about the case and the usability of the tool itself followed. At the end of part one, the participants filled in a basic demographic questionnaire about their demographics (age, gender), surgical experience, and experience with VR. The second part of the study was a group interview with the whole team before the surgery. The team was asked to do a group planning of the upcoming surgery, while one surgeon used the VR headset and the other ones followed on a monitor. The session was completed by an interview about the group experience with the tool. The third and last session was conducted after the surgery. The aim of this phase was to get an idea if the participants could identify any benefit of using the VR tool for surgery planning. Due to the current pandemic situation, all interviews were done using a video conferencing tool and the sessions were video recorded after consent by all participants.

The videos were analyzed by extracting statements and observations, which were organized by three experienced researchers using an affinity diagram (21) with the miro board^4^ tool. In total, 363 min (6 h and 3 min) of video material were analyzed. We also analyzed the time the participants spent on planning each case. The interviews contained one question asking the participants to rate the resectability of the tumors before and after the planning on a Likert scale 1 (not resectable) to 7 (very good resectable).

## 5. Findings

The video analysis revealed that the participants spent on average 14.3 min (SD = 3.59, median = 12 min) on planning the cases in VR. In the group setting, they spent 9 min (C1) and 10 min (C2) in VR. The qualitative results from the video analysis were clustered into two categories - the findings linked to the medical cases and the results related to usability, human-computer interaction, and the tool itself. Each of the categories was clustered in multiple subcategories. The following section is based on the structure and categories of the qualitative findings in the affinity diagram.

### 5.1. Medical Practice

This section presents the findings of all clusters related to medical aspects.

#### 5.1.1. Prior Knowledge

All participants reported being familiar with the case information, although P3 stated for C1 that they had not seen the data for 3-4 weeks. Open questions, which the participants hoped to answer by viewing the medical data and planning the surgery, where for example “How is the spatial arrangement of the blood vessels around the tumor^5^?” (P3-C2, P1-C1) or if there is enough space between the biliary tracts and the tumor tissue (P3-C2, P4-C2).

#### 5.1.2. Resectability

For C1, two participants reported an increase in their personal judgement regarding resectability after using the VR planning tool (the other two already reported the highest value). For C2, one participant raised the score but was not confident whether the 3D model fits the actual situation of the patient (more about this aspect in the following of this section), and the other two lowered their score.

#### 5.1.3. Recall

All participants reported that the 3D model from the planning was still present in their minds while performing the surgery and P3 reported that they felt “a little more confident regarding the blood vessels.” P1 stated that the 3D model is more vivid in memory than the standard images. Yet, P4 noted that the surgeries followed standard procedures but that they could go in with “less stress” and “more focus.”

#### 5.1.4. Added Value

In general, the participants agreed that the VR planning tool has an added value for surgery planning. P1 even said, “I'm convinced that it [planning with 3D models in VR] is extremely important and might lead to faster results with less complications intraoperatively.” P3 stated that it increases the safety in his/her opinion. While for C1 the marked resection volume fitted the expectations of P1 and P4, P3 stated beforehand that they would have expected a bigger loss of tissue for the patient. For C2, there were discussions in the group setting about how much of the liver had to be removed, and already in the individual planning session, P1 was surprised by the amount of marked volume. But P1 also said after planning with the model “this I would do differently based on the model […].” One benefit mentioned by all participants was that, in VR with the 3D model, it is easier to understand the distances and spatial relationships between the different relevant structures. P1 for example said, “One immediately has an impression where and how deep which vessels are. […] And if they are cut [during the surgery], it is bleeding terribly.” P4 said about C2 that it is easier to differentiate biliary tracts and vessels, while P3 said about C1 that they now see that “The bigger tumor has direct contact to the liver vein.” About C1, P4 mentioned that it was a good thing to know where the central liver vein was located and, especially its spatial relationship to the tumor. One benefit of the VR tool that was repeatedly highlighted by P1 but also mentioned by P3 and P4 is that the surgeon can see anatomical variants very quickly, which has a significant impact on the surgery. C1, for example, had according to P1 a rare variation that is not easy to detect in standard imaging. C2 had a variation in the biliary tracts, which would have surprised the surgeons during the surgery.

#### 5.1.5. Inherent Uncertainty of Imaging

The participants were critical about whether the 3D model fits the real situation in the patient (especially for C2). But also for C1, a few comments were made about the inherent uncertainty of the images and the resulting 3D model. The participants also reported that it seems to happen on a regular basis that there is a mismatch / radiological error between the MRI images and the intraoperative situation. As a consequence, P1 and P4 wanted to check the complete set of MRI images again after our planning session, as just one set was implemented within our VR tool.

#### 5.1.6. Workflow Integration

All participants saw so much benefit in the system, that they wanted to integrate the system into their standard workflow. After finishing C1, the participants, who already knew about C2, explicitly stated that they wanted to plan C2 using the system as well. P1, P2, and P4 especially saw a benefit for complex cases. P1 even stated, “I can easily imagine that by using this way of planning, some cases get curative.” P3 saw the potential for more patient-oriented and precise surgeries using this way of preparation. P4 also saw potential in using the system for discussing upcoming non-trivial surgeries with less experienced colleagues. In addition, the participants envisioned using the tool in settings like interdisciplinary tumor conferences, indication discussions, or radiological meetings.

### 5.2. Usability

This section presents the findings from all clusters, which were categorized under the common theme of usability.

#### 5.2.1. Ease of Use

The participants rated the system overall as “easy and intuitive” (P1, P2, P4) or “awesome” (P3). The observations of the participants using the systems support their claims, as they quickly got used to the system and, with a few questions, they used all functions to plan the surgery. This was especially salient with P4, who just got a very quick introduction due to the limited time available. All participants rejected a refresh of the introduction when using the tool a second time 2 weeks later.

#### 5.2.2. Increased Spatial Understanding

All participants highlighted the benefit of the 3D visualization, interaction, and the resulting understanding of the 3D arrangement of the structures. P2 said for example “I liked very much that I could view the model from different perspectives.” and P3 mentioned that the view is “better understandable than in the images.” The video analysis showed that half of the participants performed periodically (heavy) head and body movements around the model in VR while the other half primarily turned the model using the controller to achieve the desired view. Additionally, P4 commented that mental rotation tasks are not everybody's strength and by using our tool this difference gets irrelevant as the user can just turn the model. P3 and P4 further highlighted that the presented visualization is more memorable and P2 said that it might help to train understanding 2D images.

#### 5.2.3. Given Functionalities

All participants positively mentioned the functionality to mark the resection volume and thereby getting feedback on how much liver tissue they just marked. It was described as “very helpful” (P1). P1, P2, and P4 explicitly mentioned liking the sphere-shaped tool to mark the volume. The plane was described as helpful for standard procedures like hemihepatectomies (P4)—resecting half of the liver—but P1 also commented that the aim is to avoid this type of resections. P1-3 emphasized the helpfulness of the frame, which enables the view of the MRI images along with the 3D model. The possibility to separate the planning into steps was mentioned as helpful by P3 and P4 for two different use cases: (1) to explore alternatives (P3) and (2) to teach the structure of a surgery (P4). All participants used the drawing tool to mark surgery lines, also around the liver to indicate how the liver would be prepared before the actual surgery on the organ itself starts (P4). P1 and P3 preferred the drawing tool for drawing cuts over the scalpel-shaped tool available for volume marking and P1 said that the drawing tool works “very well.” P1 stated that placing the clips was easy while P4 said it needed some training, but P1, P2, and P4 stated that clips are a meaningful functionality.

#### 5.2.4. Usability Issues

For marking the volume the cube shape was titled as “unnatural” (P3). The marking of the scalpel was described as “very light” (P4) and P1 changed the handling of the controller to achieve more precision while using it. P1 and P3 brought up that the VR headset was uncomfortable, P1 said it was too heavy especially after spending the day in the operating room looking down. Observations revealed that grabbing new clips from the spawning point at the menu was (initially) challenging for three participants, as they missed grabbing the small circle representing the clip. On the other hand, P3 gave feedback that the clips were too large to place them at the desired place in the model. The interaction concept of grabbing objects like the 3D liver model, the frame, or the clips but using a pointer to interact with the menu seemed challenging. For P3 and P4, the confusion was sometimes clearly visible and P2 remarked that some mental effort was needed. Related to this, the participants often had to switch tools to perform the planning, as moving was just possible in the default mode, which made using the scalpel-shaped tool especially “hard” for P2 as they said “one has constantly adjusted the view.” Also, observations revealed that these changes seemed to be bothersome for some participants, P1 even asked for a second controller to be able to turn the model while annotating it.

#### 5.2.5. Requested Features

P1 wished for the possibility to move and annotate the 3D model at the same time. Furthermore, P1 and P2 wished for more views like viewing segment borders or en-/disable the view of veins and arteries. P1 and P3 asked for the actual removal of the virtual liver tissue to be able to see which blood vessels have to be treated after the tumor removal. Moreover, P1 and P4 suggested that it might be helpful if the clips prompt a change in the display of the following blood vessels, e.g., darker if disconnected. P1 strongly expressed the need for a measuring tool and P2 indicated a need for such a function as well.

#### 5.2.6. Multiuser

The opinions on whether one VR headset is sufficient or more would be usefully varied between participants and study runs. The given use cases for multiple VR headsets by the participants were either for the surgical team (resulting in up to three headsets) or for teaching. In general, all participants agreed that one VR headset and streaming this view to a monitor is a good solution, P1 suggested, that—if necessary—the VR headset could be passed on. For C2, which was a more complex case, P1 and P3 noted that a bigger screen than a 21” monitor used here “would be good.” P3 had reservations regarding using multiple VR headsets, one aspect that might lead to a negative experience in P3's opinion is that it “will be confusing” having multiple people in the VR planning on the same model.

## 6. Discussions and Limitations

Overall, the feedback from all surgeons was highly positive. The surgeons valued the tool for its easy interaction with the medical data, the resulting quick grasp on the essential individual aspects of the patient, and the good recollection of the seen data afterward.

Some of the statements from the category *Added Value* regarding the medical aspects suggest that surgical planning in VR might lead to shorter surgeries with less complications and benefits the safety of the surgery. This aspect can be supported by the fact that participants stated that they could easier see (uncommon) anatomical variants. In addition, even though all surgeons were familiar with the case data beforehand, they all reported that they found new information in the visualizations by interacting with them in VR. All surgeons also reported a better spatial understanding of the case and better impressions of distances between critical structures, refer to category *Increased Spatial Understanding*. These aspects might also play an important role in the argument of increased safety of the surgery. Improved spatial understanding might lead to more awareness of the positions and depth of blood vessels or other supporting structures as stated by P1 and P3. Avoiding damage to the major structures visible in imaging techniques (and, therefore, in the model) is critical as fixing them is either hard or impossible. Discovering anatomical variants during the operation increases the risk and surgery duration (according to P1). Therefore, a clear understanding of the upcoming situation and knowing where to be careful to avoid potentially critical situations might also lead to less stressed surgeons (as mentioned by P4) possibly having a positive impact on the surgery outcome. Furthermore, for both patients an atypical resection was planned to keep as much liver tissue as possible intact. The participants mentioned, that these procedures rely more on knowledge of the individual blood vessel structure, as hemihepatectomies follow a given guideline and are “less” complex in this aspect.

The anatomical model itself already presents a huge benefit compared to the 2D images as it is displayed as a 3D structure compared to the stack of individual images. Furthermore, the different structures are highlighted in different colors and borders are clearly visible. This improved form of information visualization alone can bring clear advantages to the surgical planning process. Nevertheless, our findings indicate that seeing these models in VR and interacting with them through virtual hands might have an even bigger advantage. The fact that the surgeons reported better spatial understanding and described the data as more memorable, which is attributable (1) to the stereoscopic view of VR as shown by McIntire et al. (6) and (2) the ability to interact with the data themselves. Directly interacting with the data yourself has been shown to benefit understanding and learning of anatomy (22). Also, the embodiment that arises from the interaction in VR is linked to improved implicit learning (23) which could transfer to the implicit learning of the individual anatomical structure and, therefore, an easier recall during the surgery. These concepts can also be found in the observation that all surgeons used head movements and rotated the model extensively to get a particular view of the model they were interested in. They also reported the ability to view the model from different angles and the ability to rotate the models with their hands as one of the most important features of the tool. While we cannot clearly distinguish the advantages of the model alone and the advantages added by the VR and its interactive nature, the observations and statements of the surgeons clearly suggest that VR brings substantial additional value. The fact that the interaction, in general, was perceived as easy and intuitive indicates that the design choice of being able to interact with all components with only one button and a flat menu structure worked well and contributed to the overall positive impression.

It has to be noted that there is inherent uncertainty in all imaging techniques and their interpretation on which the model is based, which was also mentioned by the participants, refer to category *Inherent Uncertainty of Imaging*. Therefore, by using a VR planning tool like the one presented here, the users always have to be aware that there could be a mismatch between the model and the real situation. The frame function of the application makes the users aware of this possible interpretation mismatch as they can overlay the model and the MRI or CT images.

The participants stated that in a group scenario one VR headset is sufficient when the others can follow on a (big enough) screen, refer to category *Multiuser*. These statements have to be interpreted with caution as all participants already interacted with the 3D model at hand individually before and stated that it is vivid in their memory. Therefore, they already had a good understanding of the spatial arrangement of the relevant structures when discussing the case in a group with limited information on the 2D screen. In the proposed settings, like interdisciplinary tumor conferences, the other participants would not have had the benefits provided by VR of interacting with the data firsthand and adjusting their own mental model to a common ground. Therefore, we assume that more VR headsets in a group setup would create a benefit (as also mentioned by the participants for a teaching scenario). Training or briefing a less experienced colleague in a multi-user setup could benefit from the mentioned advantages as well and expand the field of application. The work by Gloy et al. (24) already indicates, that using VR for training or teaching purposes could lead to better results in recall.

However, it should be emphasized that all surgeons want to integrate the tool into their standard workflow despite the mentioned disadvantages like the weight of the headset and slight technical problems like tracking errors. It seems that the good usability of 3D manipulation and the gathered valuable information about the patient together with the improved recall make the tool very compelling. This is also in line with results from other studies introducing VR to surgeons, e.g., the ones by Sousa et al. (18), King et al. (25), and Boedecker et al. (26). The surgeons in the study by Sousa et al. (18) were eager to integrate such a VR based tool into their daily work. The usability was also mentioned as being good in all three studies (18, 25, 26). Generally it seems like, the medical experts in these studies are very open to this technology for diagnosing, planning and training. Boedecker et al. (26) also discuss in their work the positive impact a VR system can have on surgical planning. Systems incorporating AR, where in contrast to VR the surroundings are visible and therefore often used as intraoperative navigation, also achieve positive results as in the surgeons are open to these systems and see a great benefit (7, 27, 28). Nevertheless, the systems are mostly still in the phase of feasibility testing and this is rarely done in a real setting like in this pilot study.

The surgeons spent on average 14.3 min planning the surgeries, which could further decrease when used on a daily basis and no think-aloud protocol is used. Compared to the possible benefits of improved safety this form of surgical planning provides a good value for the brief planning time.

## 7. Conclusion and Future Work

This study presents an evaluation of a VR surgical planning tool. Four surgeons used the tool to plan two real liver surgeries. Experienced surgeons described the tool as easy to use and valued the immediate access to the patient's individual anatomy using natural gestures like head movement. Consequently, they could identify surgical relevant anatomical variants faster and all participants directly wanted to integrate it into their standard workflow. With this first small study, we demonstrate the potential that VR applications and related concepts of embodiment and natural interaction can bring to the professional context, especially in the medical domain. In future iterations of the software, we will address the issues mentioned by the surgeons, e.g., make the clips easier to grab and integrate additional features like a measuring tool and the possibility to cut away parts of the organ, which should be removed. In addition, we will integrate the feature, that a clipped vessel will result in a change, indicating which parts of the liver need to be removed due to a missing connection to the major blood vessels. Therefore, this allows for a volume calculation of the affected region. Additionally, as the surgeons reported a better understanding of the patient's specific anatomy and a better recall of the localisation of the relevant structures, the VR tool has the potential to improve surgery and patient safety. To quantify these results, further studies with a larger number of participants are planned.

## Data Availability Statement

The raw data supporting the conclusions of this article will be made available by the authors, without undue reservation.

## Ethics Statement

The studies involving human participants were reviewed and approved by Carl von Ossietzky Universität Oldenburg Fakultät VI Medizin und Gesundheitswissenschaften Medizinische Ethikkommission, Ammerlnder Heerstr. 114–118, 26129 Oldenburg. The patients/participants provided their written informed consent to participate in this study. Written informed consent was obtained from the individual(s) for the publication of any potentially identifiable images or data included in this article.

## Author Contributions

AR, TM, DS, and TD developed the study design. DW contributed significantly to the prototype design, which was mainly developed by TM in collaboration with AR and supervised by RM. AR, TM, and DS carried out the data acquisition at the university hospital and remote (due to the pandemic). AR, TM, and TD were mainly responsible for the data analysis and the manuscript drafting. All authors revised and finalized the manuscript.

## Funding

This project has received funding from the German Federal Ministry of Education and Research (BMBF) in the grant program Gesundes Leben (healthy living) (grant no. 16SV8077).

## Conflict of Interest

The authors declare that the research was conducted in the absence of any commercial or financial relationships that could be construed as a potential conflict of interest.

## Publisher's Note

All claims expressed in this article are solely those of the authors and do not necessarily represent those of their affiliated organizations, or those of the publisher, the editors and the reviewers. Any product that may be evaluated in this article, or claim that may be made by its manufacturer, is not guaranteed or endorsed by the publisher.

## Footnotes

^1^ https://uk.medical.canon/clinical-solutions-oncology/oncology-hii/

^2^ https://www.microsoft.com/de-de/hololens/buy

^3^ https://www.siemens-healthineers.com/en-us/medical-imaging-it/advanced-visualization-solutions/syngovia-cinematic

^4^ miro.com

^5^All statements are translated to English from the interviewer's mother tongue.
